# Comparison of biological activities of human antithrombins with high-mannose or complex-type nonfucosylated N-linked oligosaccharides

**DOI:** 10.1093/glycob/cww001

**Published:** 2016-01-07

**Authors:** Tsuyoshi Yamada, Yutaka Kanda, Makoto Takayama, Akitoshi Hashimoto, Tsutomu Sugihara, Ai Satoh-Kubota, Eri Suzuki-Takanami, Keiichi Yano, Shigeru Iida, Mitsuo Satoh

**Affiliations:** 2 Bio Process Research and Development Laboratories, Production Division, Kyowa Hakko Kirin Co., Ltd., Takasaki-shi, Gunma 370-0013, Japan; 3 Fuji Research Park, R&D Division, Kyowa Hakko Kirin Co., Ltd., Suntou-gun, Shizuoka 411-8731, Japan; 4 Tokyo Research Park, R&D Division, Kyowa Hakko Kirin Co., Ltd., Machida-shi, Tokyo 194-8533, Japan; 5 R&D Planning Department, R&D Division; 6 Immunology & Allergy R&D Unit, R&D Division, Kyowa Hakko Kirin Co., Ltd., Chiyoda-ku, Tokyo 100-8185, Japan

**Keywords:** anticoagulant activities, antithrombin, heparin-binding affinity, high-mannose-type N-linked oligosaccharides, pharmacokinetics

## Abstract

The structure of the N-linked oligosaccharides attached to antithrombin (AT) has been shown to affect its anticoagulant activity and pharmacokinetics. Human AT has biantennary complex-type oligosaccharides with the unique feature of lacking a core fucose, which affects its biological activities by changing its heparin-binding affinity. In human plasma, AT circulates as a mixture of the α-form bearing four oligosaccharides and the β-form lacking an oligosaccharide at Asn135. However, it remains unclear how the immature high-mannose-type oligosaccharides produced by mammalian cells affect biological activities of AT. Here, we succeeded in directly comparing the activities between the high-mannose and complex types. Interestingly, although there were no substantial differences in thrombin inhibitory activity, the high-mannose type showed higher heparin-binding affinity. The anticoagulant activities were increased by heparin and correlated with the heparin-binding affinity, resulting in the strongest anticoagulant activity being displayed in the β-form with the high-mannose type. In pharmacokinetic profiling, the high-mannose type showed a much shorter plasma half-life than the complex type. The β-form was found to have a prolonged plasma half-life compared with the α-form for the high-mannose type; conversely, the α-form showed a longer half-life than the β-form for the complex-type. The present study highlights that AT physiological activities are strictly controlled not only by a core fucose at the reducing end but also by the high-mannose-type structures at the nonreducing end. The β-form with the immature high-mannose type appears to function as a more potent anticoagulant than the AT typically found in human plasma, once it emerges in the blood.

## Introduction

Human antithrombin (AT) is a serine protease inhibitor consisting of 432 amino acids; it inactivates several enzymes of the coagulation system and is produced by the liver and vascular endothelial cells. The normal concentration in human blood is high at ∼0.2 mg/mL, and the half-life is ∼3 days (Collen et al. 1977). The physiological target proteases are those of the contact activation pathway (formerly known as the intrinsic pathway), namely the activated forms of factors X (Xa), IX (IXa), XI (XIa), XII (XIIa), and to a greater extent, factor II (thrombin) (IIa). The activated form of factor VII (VIIa) from the tissue factor pathway (formerly known as the extrinsic pathway) is included as a target (Persson et al. 2001). In addition, AT inactivates kallikrein and plasmin, also involved in blood coagulation, and certain other serine proteases that are not involved in coagulation, such as trypsin and the enzyme C1 in the classical complement pathway. Protease inactivation is a consequence of trapping of the protease in an equimolar complex with AT, forming an AT-protease complex involving an interaction between the protease and a specific reactive peptide bond within AT between Arg393 and Ser394 (Olson and Björk 1994).

**Fig. 1.**
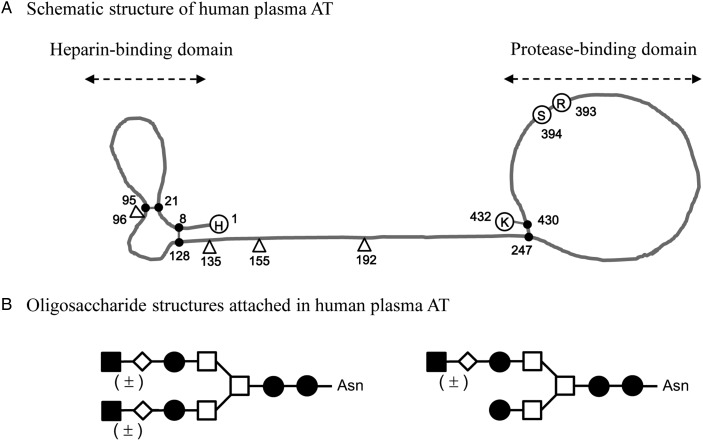
Structure of AT in human plasma. (**A**) Schematic structure of human plasma AT. Human AT contains four glycosylation sites (open triangle) and three disulfide bonds (closed circle) forming two independent protein moieties, designated as heparin- and protease-binding domains. The number represents the amino acid sequence of human AT from 1 to 432. H, His; R, Arg; S, Ser; K, Lys. (**B**) Schematic of oligosaccharide structures attached to human plasma AT based on previous reports (Franzén et al. 1980; Mizuochi et al. 1980). Biantennary complex-types composed of a mannosyl-chitobiose core structure without a core fucose were detected in human AT. The majority (>95%) contained two galactose residues (G2) and the minor form contained one galactose (G1). Sialic acid (closed square), galactose (open diamond), GlcNAc (closed circle), and mannose (open square).

Human AT contains a total of four glycosylation sites and three disulfide bonds forming two independent protein moieties that are designated as heparin- and protease-binding domains and connected through a linker portion (Figure 1A) (Travis and Salvesen 1983; Menache et al. 1992). The α-form is the dominant (90–95%) form of AT found in human plasma and has an oligosaccharide occupying each of its four glycosylation sites. A single glycosylation site at Asn135 remains consistently unoccupied in the minor (5–10%) form of the β-form (Brennan et al. 1987). The oligosaccharide structure attached to human plasma AT is a biantennary complex-type composed of a mannosyl-chitobiose core structure without a core fucose (Figure 1B) (Franzén et al. 1980; Mizuochi et al. 1980). The heparin-binding affinity of the β-form is higher than that of the α-form, which suggests that the attachment of an oligosaccharide at Asn135 hampers the binding to heparin (Turk et al. 1997; McCoy et al. 2003; Martínez-Martínez et al. 2012). Its anticoagulant activity, resulting from AT's inhibition of proteases, is increased up to several thousand fold by binding to heparin, and the heparin binding is affected by a core fucose at the reducing end of the attached N-linked oligosaccharides (Fan et al. 1993; Garone et al. 1996).

Recombinant techniques have made it possible to precisely analyze the character of human AT, avoiding complicate purification process form human blood, including other proteases and anticoagulation factors, and contamination of the pre-latent and latent inactive forms. Several studies of biological activities of recombinant human ATs (rhATs) produced by mammalian cells have been reported. In baby hamster kidney and Chinese hamster ovary cells, the oligosaccharide structures attached to these rhATs are a mixture of the biantennary complex type with or without a core fucose, and relatively lower heparin-binding affinity has been shown in the enriched fraction of rhATs with a core fucose (Fan et al. 1993; Garone et al. 1996; Mochizuki et al. 2005). The fucosylation of the oligosaccharide at Asn155 has been found to be one of the reasons for the reduction in heparin-binding affinity (Garone et al. 1996; Olson et al. 1997). The production of rhATs by the budding yeast strain *Pichia pastoris* also has been reported. The N-linked oligosaccharides of the rhATs produced by *P. pastoris* are mainly of the high-mannose type (Man9, Man10, Man11 and Man12) and additional O-linked mannosylation is found at Thr386, which is located near the hinge region of the reactive center of AT (Mochizuki et al. 2001; Hirose et al. 2002). The heparin-binding affinity of the yeast-derived rhAT is 10-fold higher than that of plasma-derived human AT (phAT). However, the O-linked mannosyl structure decreases the thrombin inhibitory activity of yeast-derived rhAT to half of that of phAT due to steric hindrance of the reactive center, which hampers understanding the influence of the high-mannose-type N-linked oligosaccharides on its activity. Recently, as one of the alternatives for phAT, rhAT produced by transgenic goats in milk has been approved for the prophylaxis of venous thromboembolism during surgery of adult patients with CAD in the EU, and for the prevention of peri-operative and peri-partum thromboembolic events in CAD in the USA (Edmunds et al. 1998; Paidas et al. 2014). In the rhAT produced by transgenic goats, the predominant oligosaccharide structure is of the monosialylated and core-fucosylated biantennary complex type, and it contains oligomannose- and hybrid-type oligosaccharides at Asn155 (Edmunds et al. 1998). The biological activity of the rhAT produced by transgenic goats is quite different from that of phAT due to its unusual oligosaccharide structures; it has a 4-fold higher heparin-binding affinity and much shorter serum half-life compared with those of phAT (Dickneite 2008). These observations show that the physiological activities of human AT are precisely controlled by its oligosaccharide structures, and illustrate the difficulty of generating an rhAT equivalent of phAT.

The glycosylation pattern is well known to change from the mature to the immature form in both physiological and pathological conditions. Constitutive patterns of protein synthesis and glycosylation are severely disrupted by acute heat stress (Henle et al. 1993). Furthermore, treatment of human blood cells with antifungal agent alters the glycosylation process and results in the accumulation of high-mannose-type glycoproteins (Frey and De Maio 2009), and pathological conditions leads to altered glycosylation patterns in some tissues (Durand and Seta 2000; Noda et al. 2003; Bernardi et al. 2013). Glycosylation inhibitors have also been found in plants, such as the sugar derivative deoxynojirimycin, which can lead to immature oligosaccharide structure formation in treated mammalian cells (Elbein 1984).

In the present study, we focused on the change in oligosaccharide structure of human AT recombinantly produced in mammalian cells from the original mature complex-type lacking a core fucose to the immature high-mannose type. CHO cell lines deficient in α-1, 6-fucosyltransferase (*FUT8*) (Yamane-Ohnuki et al. 2004) and *N*-acetylglucosaminyltransferase I (*GnT-I*) (Stanley and Chaney 1985) were employed to generate homogeneous rhATs fully lacking a core fucose with complex- (rhAT-Com) and high-mannose-type (rhAT-Man) oligosaccharides, respectively. The α-forms of the two types (rhAT-Comα and rhAT-Manα) were separated from the β-forms (rhAT-Comβ and rhAT-Manβ) by heparin-affinity chromatography, followed by anion-exchange and hydroxyapatite chromatography. The pharmacokinetics and physiological activities, including heparin binding, thrombin inhibition and anticoagulation, of these four rhATs in humans were compared in side-by-side experiments.

## Results

### Generation of homogeneous rhATs fully lacking a core fucose with high-mannose- and complex-type oligosaccharide structures

**Fig. 2.**
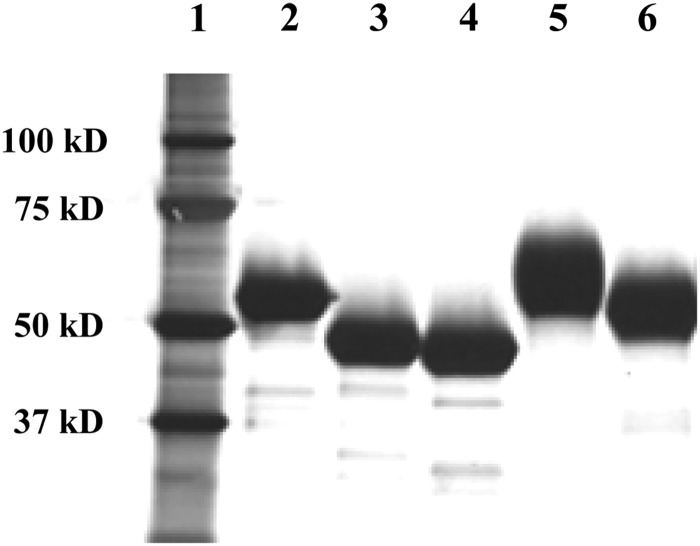
SDS–PAGE of the purified rhATs. The purified rhATs were reduced with dithiothreitol and 2 µg of each sample was run in the gels. Electrophoresis was performed in the presence of sodium dodecyl sulfate, and the samples were detected by silver staining. Protein standards (lane 1), phAT (lane 2), rhAT-Manα (lane 3), rhAT-Manβ (lane 4), rhAT-Comα (lane 5) and rhAT-Comβ (lane 6) were analyzed.

CHO cell lines lacking *FUT8* and *GnT-I* were employed to generate rhATs fully lacking a core fucose with complex-type (rhAT-Com) and high-mannose-type (rhAT-Man) oligosaccharides, respectively. The homogeneous α-forms of the two rhATs bearing four oligosaccharides (rhAT-Comα and rhAT-Manα) were separated from the β-form lacking an oligosaccharide at Asn135 (rhAT-Comβ and rhAT-Manβ) by heparin-affinity chromatography, followed by anion-exchange and hydroxyapatite chromatography as described previously (Karlsson and Winge 2003; Mochizuki et al. 2005). The aggregates and latent forms were successfully removed by heparin-affinity and anion-exchange chromatography, and they were present at <2% in each purified rhAT as determined by size-exclusion chromatography and hydrophobic interaction chromatography (Supplementary data, Table SI). The cross contamination of each form was confirmed to be at undetectable levels using hydroxyapatite chromatographic analysis (Supplementary data, Table SII). Each purified product yielded one major band in sodium dodecyl sulfate–polyacrylamide gel electrophoresis (SDS–PAGE) under reducing conditions, with quality equivalent to that of phAT (Figure 2). The rhAT-Man showed high electrophoretic mobility compared with that of rhAT-Com. The latent form of rhAT (rhAT-L) was also prepared from the side fraction.

Monosaccharaide composition analysis confirmed that there were no fucose residues in all of the purified rhATs and that there were no saccharides detected except mannose and GlcNAc in the rhAT-Man (Table I). In further oligosaccharide analysis using modified matrix-assisted laser desorption/ionization time-of-flight mass spectrometry (MALDI-TOF MS), high-mannose-type oligosaccharides consisting of five mannoses (M5) were detected in the purified rhAT-Man and sialylated biantennary complex-type oligosaccharides were detected in the purified rhAT-Com (Table I).

**Table I.** Summary of oligosaccharide analysis of the rhATs produced by CHO cells

Samples^a^	Relative composition of monosaccharides					Structure^d^
	Fucose	GlcNAc^b^	Mannose	Galactose	Sialic acid^c^	
phAT	0.03	4.0	2.3	1.9	2.0	
rhAT-Manα	n.d.	2.0	5.3	n.d.	n.d.	I
rhAT-Manβ	n.d.	2.0	5.4	n.d.	n.d.	I
rhAT-Comα	n.d.	4.0	1.9	2.1	2.3	II, III
rhAT-Comβ	n.d.	4.0	2.2	1.8	1.8	II, III

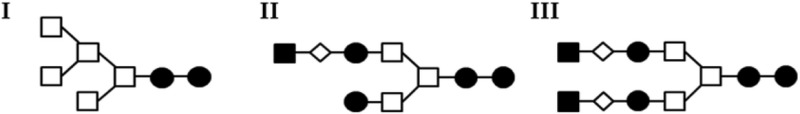

Sialic acid (closed square), galactose (open diamond), GlcNAc (closed circle) and mannose (open square).

n.d., not detected.

^a^The purified phAT, rhAT-Manα, rhAT-Manβ, rhAT-Comα and rhAT-Comβ were employed as samples.

^b^Molar ratios calculated versus GlcNAc composition.

^c^Mol sugar/oligosaccharide chain.

^d^Schematic of the major oligosaccharide structures detected by MALDI-TOF MS are described above.

### The heparin-binding affinity of rhATs

The dissociation constant (*K*_d_) for the binary complex of heparin and each of the purified rhATs (rhAT-Manα, rhAT-Manβ, rhAT-Comα and rhAT-Comβ) was determined. The data shown are the means ± SE of triplicate experiments (Table II). The rhAT bearing the high-mannose type displayed a higher heparin affinity (smaller *K*_d_) than the rhAT bearing the complex type. Actually, the rhAT-Comα had a 9-fold larger *K*_d_ compared with that of rhAT-Manα, and rhAT-Comβ had a slightly larger *K*_d_ than that of rhAT-Manβ. The heparin-binding affinity of the β-form was higher than that of the α-form, irrespective of the oligosaccharide structures. A greater heparin-binding affinity enhancement (8.6-fold) between the α- and β-forms was observed in the complex-type rhATs comparing with that (1.8-fold) of the high-mannose-type. Consequently, the order of the heparin-binding affinity of the purified rhATs was rhAT-Comα < rhAT-Comβ ≈ rhAT-Manα < rhAT-Manβ.

**Table II.** The heparin-binding affinity of the rhATs produced by CHO cells

Samples^a^	*K*_d_ (nM)	SE
rhAT-Manα	2.4	0.1
rhAT-Manβ	1.3	0.2
rhAT-Comα	21.5	2.4
rhAT-Comβ	2.5	0.4

*K*_d_, dissociation constant; SE, standard error.

^a^The purified rhAT-Manα, rhAT-Manβ, rhAT-Comα and rhAT-Comβ were employed as samples.

### The heparin cofactor activity of rhATs

**Fig. 3.**
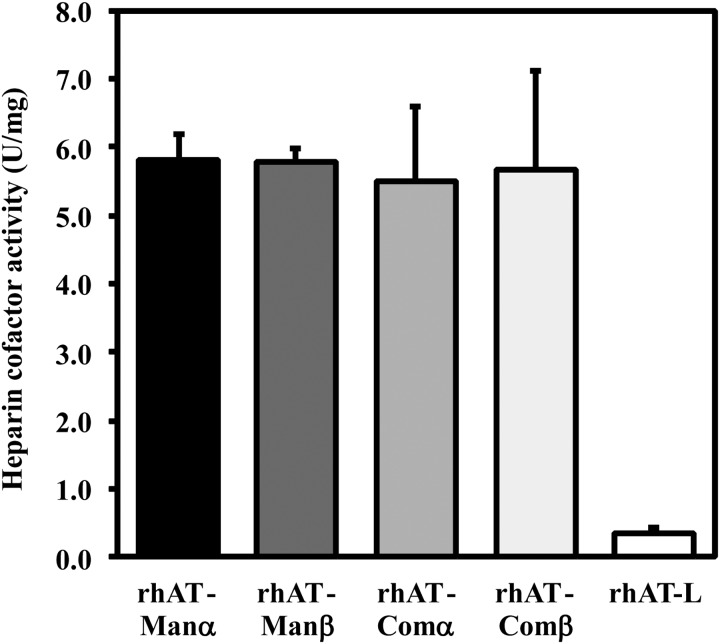
The heparin cofactor activity of the purified rhATs. The heparin cofactor activity was measured at the end of thrombin inactivation in the presence of heparin. The data shown are the means ± SD of triplicate experiments. The rhAT-Manα, rhAT-Manβ, rhAT-Comα, rhAT-Comβ and rhAT-L were employed as samples. No significant differences observed between rhAT-Manα, rhAT-Manβ, rhAT-Comα and rhAT-Comβ, as determined by Dunnett's test (*P* < 0.05).

The heparin cofactor activity, demonstrating the total active AT content as protease inhibitor, was measured as the residual thrombin activity after completion of the reaction between AT and thrombin in the presence of heparin. The data shown are the means ± SD of triplicate experiments (Figure 3). There were no significant differences observed in the heparin cofactor activity among the purified rhATs (rhAT-Manα, rhAT-Manβ, rhAT-Comα and rhAT-Comβ). rhAT-Man retained an activity comparable with that of rhAT-Com, whereas the latent inactive form of rhAT-L showed a low heparin cofactor activity. The heparin cofactor activity (mean ± SD IU/mg) of each purified rhAT was 5.83 ± 0.43 for rhAT-Manα, 5.80 ± 0.22 for rhAT-Manβ, 5.49 ± 1.11 for rhAT-Comα, 5.66 ± 1.45 for rhAT-Comβ and 0.35 ± 0.09 for rhAT-L, respectively.

### The thrombin inhibition kinetics of rhATs

To confirm the AT activity as a protease inhibitor, the inhibition of thrombin by the purified rhATs (rhAT-Manα, rhAT-Manβ, rhAT-Comα and rhAT-Comβ) was measured by calculating the second-order rate constant of the reaction in the presence or absence of heparin. The data shown are the means ± SD of triplicate experiments (Table III). In the absence of heparin, the rhAT-Comβ showed a slightly higher second-order rate constant than the other purified rhATs. The mechanism by which the structure of rhAT-Comβ affects the thrombin inhibitory reaction rate remains to be elucidated. In the presence of heparin, the thrombin inhibitory rate dramatically increased up to 2500- to 3000-fold in the range of 1.9 × 10^7^ to 2.0 × 10^7^/M/s, and there were no significant differences observed in the second-order rate constant among the purified rhATs as a result.

**Table III.** The thrombin inhibitory activity of the rhATs produced by CHO cells

Samples^a^	Second-order rate constant			
	Without heparin		With heparin	
	(/M/s)	SD	(/M/s)	SD
rhAT-Manα	6.6 × 10^3^	1.1 × 10^2^	1.9 × 10^7^	2.2 × 10^5^
rhAT-Manβ	6.1 × 10^3^	6.7 × 10	1.9 × 10^7^	5.9 × 10^5^
rhAT-Comα	6.2 × 10^3^	1.6 × 10^2^	1.9 × 10^7^	1.2 × 10^5^
rhAT-Comβ	7.3 × 10^3^	3.3 × 10^2^	2.0 × 10^7^	1.2 × 10^5^

SD, standard deviation.

^a^The purified rhAT-Manα, rhAT-Manβ, rhAT-Comα and rhAT-Comβ were employed as samples.

### The anticoagulant activities of rhATs

**Fig. 4.**
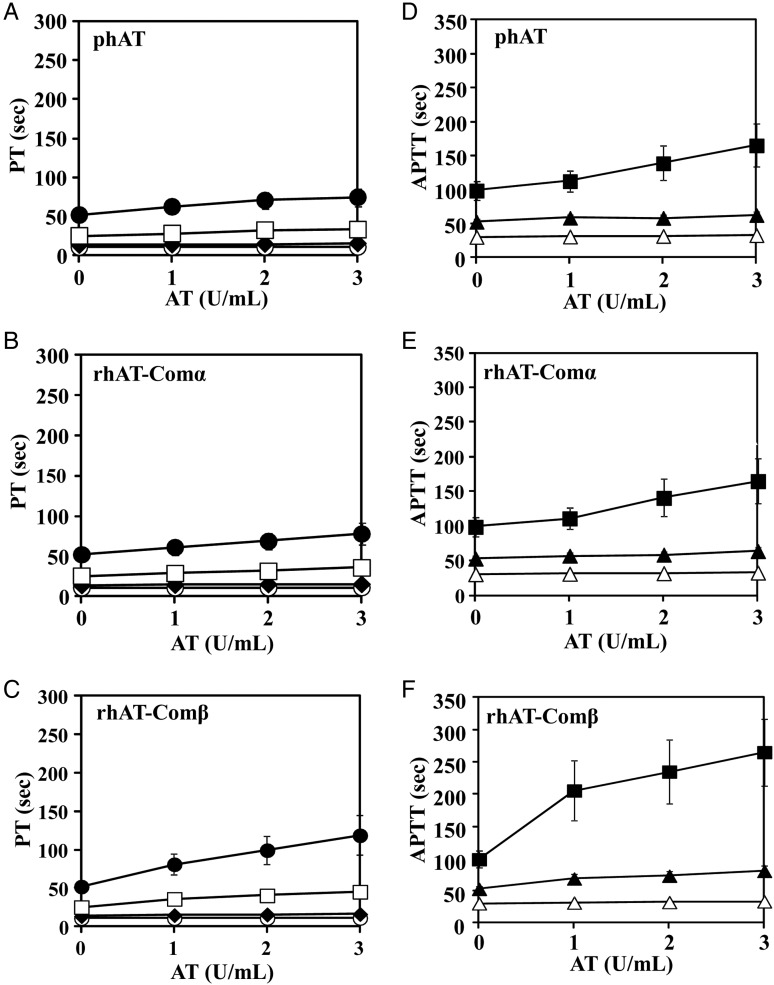
The anticoagulant responses of the purified rhAT-Com in human plasma. Human AT samples were mixed with human plasma prepared from healthy volunteers and heparin at a final concentration of 0 U/mL (open circle), 1 U/mL (closed diamond), 2 U/mL (open square) and 3 U/mL (closed circle) for PT (**A**–**C**) and 0 U/mL (open triangle), 0.2 U/mL (closed triangle), and 0.4 U/mL (closed square) for APTT (**D**–**F**). The PT and APPT were measured by an automated coagulation analyzer (Sysmex CA-5000). The data shown are the means ± SE of nine experiments. The PT of phAT (A), rhAT-Comα (B), rhAT-Comβ (C) and the APTT of phAT (D), rhAT-Comα (E) and rhAT-Comβ (F) are shown.

**Fig. 5.**
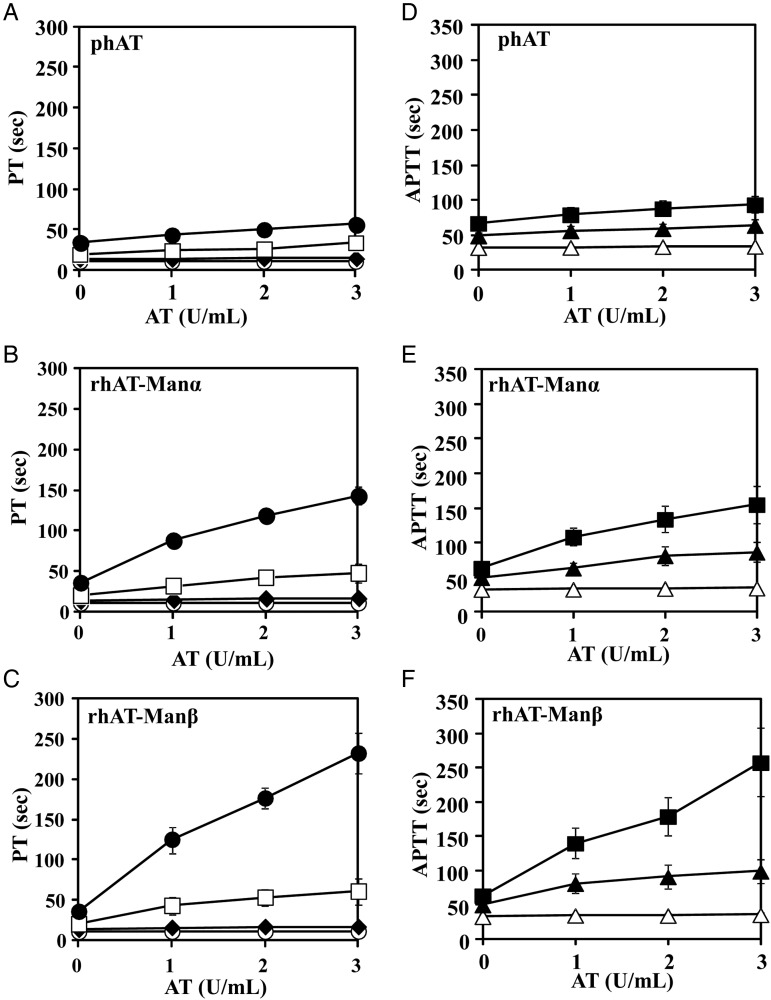
The anticoagulant responses of the purified rhAT-Man in human plasma. Human AT samples were mixed with human plasma prepared from healthy volunteers and heparin at a final concentration of 0 U/mL (open circle), 1 U/mL (closed diamond), 2 U/mL (open square) and 3 U/mL (closed circle) for PT (**A**–**C**) and 0 U/mL (open triangle), 0.2 U/mL (closed triangle) and 0.4 U/mL (closed square) for APTT (**D**–**F**). The PT and APPT were measured by an automated coagulation analyzer (Sysmex CA-5000). The data shown are the means ± SE of five experiments. The PT of phAT (A), rhAT-Manα (B), rhAT-Manβ (C) and the APTT of phAT (D), rhAT-Manα (E) and rhAT-Manβ (F) are shown.

To compare anticoagulant activities of each of the purified rhATs (rhAT-Manα, rhAT-Manβ, rhAT-Comα and rhAT-Comβ), prothrombin time (PT) and activated partial thromboplastin time (APTT) were measured in human plasma (Figure 4: rhAT-Com versus phAT, Figure 5: rhAT-Man versus phAT). In all the AT molecules, the prolongation of the PT and APTT was almost undetectable in the absence of heparin. In the presence of heparin (≥2 U/mL for PT, ≥0.2 U/mL for APTT), the PT and APTT were markedly prolonged in an AT concentration-dependent manner. The PT and APTT of the β-form were prolonged more than those of the α-form, irrespective of the complex versus high-mannose types. The rhAT-Manα exhibited a more prolonged PT and APTT than phAT, although rhAT-Comα showed a PT and APTT comparable with those of phAT. The order of the prolonged PT and APTT of the rhATs was rhAT-Comα < rhAT-Comβ ≈ rhAT-Manα < rhAT-Manβ, which well reflected the order of the heparin-binding affinity of each of the purified rhATs.

### The pharmacokinetics of rhATs

**Fig. 6.**
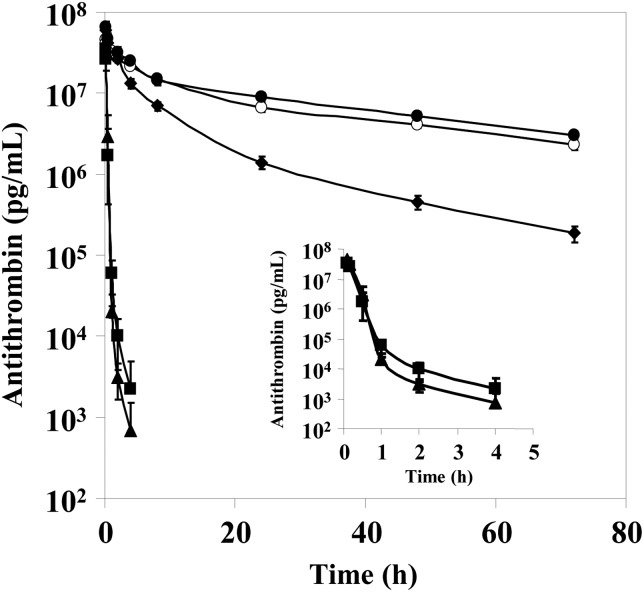
The pharmacokinetics of the purified rhATs in rabbits. Male Kbs:NZW rabbits were injected with human AT. The concentrations of the injected human AT in plasma were determined by a human AT-specific ELISA. The data shown are the means ± SD of four experiments. The rhAT-Manα (closed triangle), rhAT-Manβ (closed square), rhAT-Comα (closed circle), rhAT-Comβ (closed diamond) and phAT (open circle) were employed as samples.

To determine the pharmacokinetics, each purified rhAT (rhAT-Manα, rhAT-Manβ, rhAT-Comα and rhAT-Comβ) was injected intravenously in rabbits at a dose of 2 mg/kg body weight, and the plasma concentration of the administrated rhATs was monitored using a human AT-specific ELISA (Figure 6). The terminal half-life of rhAT-Comα was very similar to that of phAT, i.e., 31.1 versus 32.6 h, respectively. rhAT-Comβ had a terminal half-life of 16.1 h, which was half as long as that of rhAT-Comα. In contrast, the clearance of rhAT-Man was significantly faster than that of rhAT-Com, irrespective of the α- versus β-forms. Within 1 h after administration, the concentration of rhAT-Manα and rhAT-Manβ was decreased to <1/100 of the initial concentration. Interestingly, rhAT-Manβ showed a prolonged plasma half-life compared with that of rhAT-Manα, although rhAT-Comα conversely exhibited a much longer half-life than that of rhAT-Comβ.

## Discussion

Human AT is a unique glycoprotein whose oligosaccharides affect its biological activities, and is responsible for physiological homeostasis of anticoagulation. It is interesting to know the reason why such a glycostructure-sensitive glycoprotein is responsible for the maintenance of a very basic homeostatic mechanism such as anticoagulation, which directly controls life and death in humans. Human AT has biantennary complex-type oligosaccharide structures with the unique feature of lacking a core fucose via α-1,6 linkage at the reducing end (Figure 1). The core fucose affects its anticoagulant activity through changing the heparin-binding affinity (Fan et al. 1993), and is missing due to its generation by hepatocytes (Miyoshi et al. 1999). The heparin binding greatly enhances the rate of AT's inhibition of proteases, resulting in a prompt anticoagulation (Chang et al. 1996). In human plasma, AT circulates as a mixture of two forms with different numbers of attached N-linked oligosaccharides, namely the α-form bearing four oligosaccharides and the β-form bearing three oligosaccharides and lacking an oligosaccharide at Asn135. The α- and β-forms differ in their affinity for heparin, with the β-form having a higher affinity >10-fold (Turk et al. 1997; McCoy et al. 2003). Even though the β-form is present at only 5–10% of the total AT in human plasma (Brennan et al. 1987), due to its increased heparin-binding affinity, it is thought that the β-form plays a more critical role than the α-form in controlling thrombogenic events caused by tissue injury (Swedenborg 1998). Indeed, thrombin inhibition after injury to the aorta has been attributed mainly to the β-form (Frebelius et al. 1996), which means that AT with a higher heparin-binding affinity appears to be favorable for the quicker response for inhibiting excess coagulation in tissue injury emergencies. Thus, it is interesting to know which glycoform of human AT shows the strongest binding affinity for heparin.

The glycosylation patterns are well known to be different depending on host cells, and many attempts to generate rhAT have failed to show that the recombinant products are equivalent to phAT. Non-human glycosylation patterns, including a core fucose via α-1,3 linkage at the reducing end of N-linked oligosaccharides in insect cells (Gillespie et al. 1991; Ersdal-Badju et al. 1995; Tomiya et al. 2004), hyper-mannose antenna N-linked oligosaccharides and O-linked mannosylation at Thr386 of rhAT in yeast (Mochizuki et al. 2001; Hirose et al. 2002), have been observed. These unusual structures hamper understanding the physiological roles of the oligosaccharide-modified human AT, and the immunogenicity of these non-human glycosylations is also a concern for their applicability as therapeutics. Even in mammalian cells, including transgenic goats, the attachment of a core fucose via α-1,6 linkage to the N-linked oligosaccharides of rhAT has been documented, and was shown to change the biological activity through reducing its heparin-binding affinity (Fan et al. 1993; Garone et al. 1996; Olson et al. 1997; Edmunds et al. 1998; Mochizuki et al. 2005). Core fucosylation via α-1,6 linkage is solely mediated by the α-1,6-fucosyltransferase FUT8 and is widely distributed in mammalian cells, except hepatocytes (Miyoshi et al. 1999). The increased expression of *FUT8* and the extent of core fucosylation are reported to be altered under pathological conditions such as hepatocellular carcinoma and liver cirrhosis (Miyoshi et al. 1999; Noda et al. 2003; Bernardi et al. 2013), and is strongly linked to age-related changes in glycosylation in the liver (Vanhooren et al. 2007, 2010). Thus, it might be interesting to analyze how these alterations affect the physiological activities of human AT by concomitantly modifying its oligosaccharide structures. Indeed, a slight but significant amount of fucose was detected in phAT from a pool of human blood derived from several thousand volunteers (Table I).

In the rhAT generated by transgenic goats, mammalian immature oligosaccharide structures have been observed, particularly the oligomannose and hybrid types at Asn155, in addition to the monosialylated and core-fucosylated biantennary complex-type oligosaccharides (Edmunds et al. 1998). These are typical intermediate immature forms in oligosaccharide biosynthesis of mature complex-type oligosaccharides in mammalian cells. The biological activities of the rhAT produced in transgenic goats are quite different from those of phAT due to these immature oligosaccharides and the core-fucosylated complex-type oligosaccharides. Compared with phAT, the clearance of the goat-produced rhAT was reported to be seven times faster and its half-life time was nine times shorter in humans in clinical trials (Dennis et al. 2009). Interestingly, despite the fact that the majority of the oligosaccharides are the core-fucosylated biantennary complex type, the heparin-binding affinity of the goat-produced rhAT is 4-fold higher than that of phAT (Dickneite 2008). The heparin-binding affinity of human AT appears to be enhanced by attachment of the immature oligosaccharides, such as the high-mannose and hybrid types, rather than the mature complex-type modification. Our results show that the heparin-binding affinity of the high-mannose-type rhATs is higher than that of the complex-type rhATs, and that the α-form of rhAT bearing the high-mannose type has a high heparin-binding affinity comparable with that of the β-form of rhAT bearing the complex type, in spite of additional glycosylation at the Asn135 that is thought to negatively regulate the affinity in the α-form (Table II). Previous study demonstrated that the higher affinity of the β-form bearing the complex type is due to the increased rate at which subsequent conformational changes occur within the protein upon initial heparin binding, and also that the additional glycosylation at Asn135 for the α-form bearing the complex-type is not thought to interfere with initial heparin binding, but rather to slow AT conversion from the native to the activated conformations (McCoy et al. 2003). There were no substantial differences observed in thrombin inhibitory activity, including the heparin cofactor activity (Figure 3) and thrombin inhibition kinetics (Table III), which means that conversion of N-linked oligosaccharides from the complex type to the high-mannose type does not affect AT's inhibitory activity against proteases. The heparin-binding affinity of the β-form was confirmed to be higher than that of the α-form, irrespective of the oligosaccharide structures (Table II). A greater heparin-binding affinity enhancement between the α- and β-forms was observed in the complex-type rhATs compared with those of the high-mannose-type rhATs (Table II), demonstrating that the complex-type structure may sterically hinder heparin binding in AT to a greater extent than the high-mannose-type structure. To conclude the reason why the α-form with the high-mannose-type shows a heparin-binding affinity so close to that of the β-form with the complex-type despite of the additional glycosylation at Asn135, each tertiary structure should be solved. The anticoagulant activities, including those of APTT and PT in human plasma, were increased with heparin and closely correlated to the heparin-binding affinity (Figures 4 and 5). As a result, among the four generated rhATs (rhAT-Comα, rhAT-Comβ, rhAT-Manα and rhAT-Manβ), the β-form rhAT bearing the immature high-mannose-type (M5) oligosaccharide was found to have the highest heparin-binding affinity and the strongest anticoagulant activities.

In our pharmacokinetic study, rhAT bearing the high-mannose-type oligosaccharide had a much shorter serum half-life than rhAT bearing the complex type, irrespective of the α- versus β-forms (Figure 6). The β-form showed a prolonged plasma half-life compared with that of the α-form in rhAT bearing the high-mannose type, although the α-form conversely showed much longer half-life than the β-form in rhAT bearing the complex type (Figure 6). These results demonstrate that the clearance mechanisms are different depending on the oligosaccharide structures of AT. In addition to the heparin-binding affinity, the number of sialic acid residues at the nonreducing end of the attached N-linked oligosaccharides appears to contribute to the plasma half-life in the complex type (Egrie and Browne 2001). On the other hand, the number of mannose residues exposed at the nonreducing end likely affects plasma circulation time through mannose receptor-mediated uptake in the liver and macrophages (Lee et al. 2002; Kanda et al. 2006; Mi et al. 2014).

In conclusion, we have focused on the change in the oligosaccharide structure of human AT produced by mammalian cells from the mature complex-type predominantly existing in human plasma to the immature high-mannose type, and succeeded in generating homogeneous rhATs bearing high-mannose and complex N-linked oligosaccharides fully lacking a core fucose using two unique CHO cell lines deficient in *FUT8* and *GnT-I*. The direct comparison of the activities between the high-mannose and complex types has shown that AT physiological activities, including heparin-binding affinity, thrombin inhibitory activity, anticoagulant activities in human plasma and pharmacokinetics, are strictly controlled not only by a core fucose at the reducing end but also by the N-linked structure of the high-mannose type at the nonreducing end. The β-form of human AT bearing the immature high-mannose-type oligosaccharides exhibited the strongest heparin-binding affinity and appeared to function as a more potent anticoagulant than AT typically found in human plasma, once it emerges in the blood. Although the α-form of rhAT with the complex type produced by *FUT8*-knockout CHO cells is thought to be suitable for a substitute for phAT drug on the market in terms of its anticoagulant activities and pharmacokinetics, the β-form of rhAT with the high-mannose type generated by *GnT-I*-deficient CHO cells has a potential to provide a new therapy as a more potent anticoagulant.

## Materials and methods

### Materials

Human thrombin was purchased from ERL (South Bend, IN). Human plasma-derived AT approved on the market was purchased from Mitsubishi Well Pharma Co., Ltd. (Tokyo, Japan) and used as a standard for phAT. Human plasma was obtained from healthy volunteers. Blood donors were randomly selected among the volunteers registered at Tokyo Research Park, Kyowa Hakko Kirin, Co., Ltd. All donors gave written informed consent before the analyses.

### Cell lines

The CHO cell line lacking endogenous *GnT-I*, Pro-5WgaRI3C (Lec1) (Stanley and Chaney 1985), was purchased from the American Type Culture Collection (ATCC, Manassas, VA). The *FUT8*-knockout CHO cell line was established in our laboratory (Yamane-Ohnuki et al. 2004). Both CHO cell lines were cultured in Iscove's modified Dulbecco's medium (IMDM) containing 10% (v/v) dialyzed fetal bovine serum, 0.1 mmol/L hypoxanthine and 16 mmol/L thymidine (all from Invitrogen, Carlsbad, CA).

### Establishment of rhAT-producing cells

The vector pKAN-AT for expression of rhAT was generated as follows. Full-length human AT cDNA was isolated by PCR from a human liver cDNA library (Life Technologies Japan, Tokyo, Japan) using the primers 5′-CGGAATTCGCCACCATGTATTCCAATGTGATAGGAACTGTAAC-3′ and 5′-CGGGATCCTTACTTAACACAAGGGTTGGCTACTCTG-3′. The isolated cDNA was subcloned into the expression vector pKANTEX93 (Nakamura et al. 2000) at the *Eco*RI/*Bam*HI sites to generate the pKAN-AT. The vector was transfected into CHO cells by electroporation, and the transfectants were selected on the basis of AT production during stepwise gene amplification in IMDM containing methotrexate (Sigma-Aldrich, St. Louis, MO) from 0, 50, 200 and up to 500 nmol/L. The AT concentration in the cell culture supernatant was measured by a human AT-specific ELISA using the Matched Pair Antibody Set for ELISA of Human Antithrombin Antigen (Affinity Biologicals, Ancaster, Canada) per the manufacturer's instructions.

### Purification of the rhATs

The selected CHO transfectants were grown to confluence and cultured in EX-CELL 302 medium (JRH Biosciences, Piscataway, NJ), supplemented with 6 mmol/L l-glutamine for 5 d. The supernatant was centrifuged to remove cellular debris, and then filtered through a 0.22-µm filter. The filtered supernatant was applied to a heparin Sepharose FF column (GE Healthcare, Uppsala, Sweden), equilibrated with 50 mmol/L Tris–HCl, 14 mmol/L sodium citrate and 150 mmol/L sodium chloride (pH 7.4), and washed with the same buffer. The elution was performed with a linear gradient running from 0 to 2.5 mol/L sodium chloride in 50 mmol/L Tris–HCl, 14 mmol/L sodium citrate (pH 7.4) over 10 column volumes. The heparin column eluate was concentrated using a Biomax 10 (Millipore, Billerica, MA), exchanged with 20 mmol/L sodium phosphate (pH 7.4), loaded onto a DEAE Sepharose FF column (GE Healthcare) equilibrated with 20 mmol/L sodium phosphate (pH 7.4), and washed with the same equilibration buffer. The elution was performed with a linear gradient running from 0 to 1.0 mol/L sodium chloride in sodium phosphate (pH 7.4) over eight column volumes. The eluate was loaded onto a CHT ceramic hydroxyapatite Type-1 column (Bio-Rad, Hercules, CA) to separate the α- and β-forms of AT. The column was equilibrated with 0.01 mmol/L calcium chloride in 20 mmol/L sodium phosphate (pH 6.8) and was washed with the same equilibration buffer. The elution was performed with a linear gradient of 0–2.5 mol/L sodium chloride in 50 mmol/L Tris–HCl, 14 mmol/L sodium citrate (pH 7.4) over 10 column volumes. The eluted fractions were concentrated using a Biomax 10 and exchanged with Dulbecco's phosphate-buffered saline (pH 7.4; Invitrogen). The latent rhAT-L and the aggregates were monitored and separated primarily by heparin or hydroxyapatite chromatography.

### Purity analysis of the rhATs

The purified rhATs were analyzed by SDS–PAGE using precast 5–20% polyacrylamide Tris-glycine gels (ATTO, Tokyo, Japan), and the bands were visualized by silver staining (Cosmo Bio, Tokyo, Japan). The content of rhAT-L was analyzed by hydrophobic interaction chromatography using 50 mmol/L Tris–HCl buffer as described previously (Mochizuki et al. 2005). The aggregate contents were determined by size-exclusion chromatography on a TSK-GEL G2000SWxl column (7.5 mm I.D. × 75 mm, TOSOH, Tokyo, Japan) using a Shimadzu HPLC system (Kyoto, Japan) as follows. The mobile phase consisted of 50 mmol/L phosphate buffer and 300 mmol/L sodium chloride (pH 6.7), the flow rate was 0.5 mL/min, and the signal was monitored at an absorbance of 280 nm.

### Estimation of the α-form content in the rhATs

The content of the α-form in the purified rhATs was assessed on a CHT2-I hydroxyapatite column (7.0 mm × 50 mm I.D.; Bio-Rad) using a Shimadzu HPLC system. The column was developed with a complex gradient at a flow rate of 0.5 mL/min. Solvent A consisted of 10 mmol/L sodium phosphate and 0.01 mmol/L calcium chloride (pH 6.8), and solvent B consisted of 500 mmol/L sodium phosphate and 0.01 mmol/L calcium chloride (pH 6.8), respectively. The following gradient program was used: 0 to 5 min = 10% solvent B, 5 to 15 min = 10% to 50% solvent B, 15 to 17 min = 50% to 100% solvent B, and 17 to 27 min = 100% solvent B to separate the α-form. The protein was monitored at an absorbance of 280 nm.

### Analysis of AT-derived N-linked oligosaccharides

The monosaccharide composition of each purified rhAT was characterized by modified high-performance anion-exchange chromatography; monosaccharides were released from an aliquot of rhAT by heating with 4 mol/L trifluoroacetic acid at 100°C for 2 h and dried under a vacuum. The monosaccharides reconstituted in sterile distilled water were analyzed using a waveform and DX500 system (DIONEX, Sunnyvale, CA). A CarboPac PA-1 column (DIONEX) was used to resolve monosaccharides in 18 mmol/L sodium hydroxide solution with a flow rate of 0.8 mL/min at 35°C as described previously (Shinkawa et al. 2003). Sialic acid was determined using a Sialic Acid Fluorescence Labeling Kit (Takara Bio, Shiga, Japan) per the manufacturer's instructions.

The oligosaccharide profile of the purified rhATs was characterized by MALDI-TOF MS. The rhATs were treated using glycan purification kit (BlotGlyco; Sumitomo Bakelite, Tokyo, Japan) according to the manufacturer's protocol. Briefly, 1 mg of rhATs were denatured and trypsinized, and their N-linked oligosaccharides were released from the peptides by treating with peptide-*N*-glycosidase F (PNGaseF; Sigma-Aldrich, St. Louis, MO). The released glycans were captured by BlotGlyco beads, and sialic acid residues of *N*-glycans were methyl esterified to stabilize them in the mass spectrometer. The captured glycans were released in derivatized form with a labeling reagent, and the aliquots of labeled *N*-glycan were spotted onto a MALDI target plate. MALDI-TOF MS analysis was performed on an Ultraflex III mass spectrometer (Bruker Daltonics, Bremen, Germany) in the positive-ion, reflectron mode.

### Heparin-binding affinity

The heparin-binding affinity of the purified rhATs was determined by the increase in tryptophan fluorescence accompanying the interaction using a fluorophotometer (RF-5300PC; Shimadzu). The titrations were performed at 25°C in 20 mmol/L sodium phosphate, 100 mmol/L sodium chloride, 100 nmol/L EDTA and 0.1% PEG 6000 (pH 7.4). The excitation and emission wavelengths were 280 (±1.5 nm) and 340 nm (±5.0 nm), respectively. The dissociation constant (*K*_d_) was analyzed as previously described (Olson et al. 1993; Jairajpuri et al. 2002) using GraphPad Prism 4 (GraphPad, Inc., La Jolla, CA).

### Heparin cofactor activity

The heparin cofactor activity was measured at the end of thrombin inactivation in the presence of heparin as described previously (Abildgaard et al. 1977). The purified rhATs were incubated with 2.5 U/mL human thrombin in 50 mmol/L Tris–HCl, 0.15 mol/L sodium chloride and 0.2% bovine serum albumin (pH 8.3) at 37°C for 5 min in the presence of 0.6 U/mL heparin. The residual thrombin activity was assayed by incubating with 2.0 mmol/L S-2238 (Sekisui Medical, Tokyo, Japan) as a substrate at 37°C for 2 min, and was measured at an absorbance of 405 nm. As a standard, phAT (5.88 IU/mg) was employed to estimate the value of heparin cofactor activity.

### Thrombin inhibition kinetics

The kinetics of thrombin inhibition of the purified rhATs in the absence or presence of heparin was measured as follows. The thrombin inhibition kinetics of the rhATs were measured at 25°C in 20 mmol/L sodium phosphate, 100 mmol/L sodium chloride, 100 µmol/L EDTA and 0.1% polyethylene glycol (pH 7.4). The ATs (at a final concentration of 100 nmol/L) were mixed with thrombin (at a final concentration of 1–10 nmol/L) in the presence or absence of heparin (at a final concentration of 50 pmol/L), and were allowed to react for 1–25 min. The concentration of residual thrombin was assessed by the hydrolysis rate of S-2238 and the pseudo-first-order rate constants were determined. The second-order rate constants for the inhibition of thrombin by the rhATs in the absence or presence of heparin were obtained from the pseudo-first-order rate constants as previously described (Olson et al. 1993; Jairajpuri et al. 2002).

### Anticoagulant activities of rhATs

The anticoagulant activities, including APTT and PT in human plasma, of the rhATs were measured using an automated coagulation analyzer (Sysmex CA-5000; Sysmex, Hyogo, Japan) according to the procedures recommended by the manufacturer. Briefly, human blood obtained from healthy volunteers was gently mixed with 1/10 volume of 3.8% (w/v) sodium citrate and centrifuged at 1300 × *g* for 15 min at 4°C to separate human plasma. The purified rhATs were diluted to a concentration of 3 IU/mL. Then, 20 µL of prepared ATs were mixed with 170 µL of the human plasma and 10 µL heparin solution, and then PT and APTT were measured using Thromboplastin C Plus (Sysmex) as detection reagent for PT, and Datafai APTT (Sysmex) and 0.02 mmol/L calcium chloride solution (Sysmex) as detection reagents for APTT.

### Pharmacokinetic analysis of rhATs

Male Kbs:NZW rabbits (1.5–2.0 kg; Kitayama Labes Co., Ltd., Nagano, Japan) were used for the AT clearance study. The rabbits were injected with 2.0 mg/kg body weight of the purified rhATs via the auricular ear vein. Blood samples were withdrawn from the auricular vein of the opposite ear, and were drawn into 1/10 volume of 3.8% (w/v) sodium citrate. The concentrations of the rhATs were determined by a human AT-specific ELISA using a mouse anti-human AT antibody (US Biological, Swampscott, MA) and a sheep anti-human AT peroxidase-conjugated antibody (Affinity Biologicals). The pharmacokinetic parameters were obtained by a two-compartment analysis program using the WinNonlin Professional software (version 4.1; Pharsight, Mountain View, CA). All animals were maintained at 20–24°C under a 12-h light/dark cycle and were maintained in compliance with the guidelines formulated by the Japanese Pharmacological Society. The protocol was approved by the Bioethical Committee of the Pharmaceutical Research Center, Kyowa Hakko Kirin Co., Ltd. (protocol number: 08-265).

## Supplementary Data

Supplementary data for this article are available online at http://glycob.oxfordjournals.org/.

## Funding

This study was carried out without funding. Funding to pay the Open Access publication charges for this article was provided by Kyowa Hakko Kirin Co., Ltd.

## Conflict of interest statement

None declared.

## Abbreviations

APTT, activated partial thromboplastin time; AT, antithrombin; *FUT8*, α-1,6-fucosyltransferase; *GnT-I*, *N*-acetylglucosaminyltransferase I; IMDM, Iscove's modified Dulbecco's medium; *K*_d_, dissociation constant; MALDI-TOF MS, matrix-assisted laser desorption/ionization time-of-flight mass spectrometry; rhAT, recombinant human AT; rhAT-Com, rhAT bearing sialylated biantennary complex-type N-linked oligosaccharides lacking a core fucose; rhAT-Comα, α-form of rhAT-Com; rhAT-Comβ, β-form of rhAT-Com; rhAT-L, latent form of rhAT; rhAT-Man, rhAT bearing high-mannose-type N-linked oligosaccharides; rhAT-Manα, α-form of rhAT-Man; rhAT-Manβ, β-form of rhAT-Man; SDS–PAGE, sodium dodecyl sulfate polyacrylamide gel electrophoresis; phAT, plasma-derived human AT; PT, prothrombin time.

## Supplementary Material

Supplementary Data
