# Supplementary material for: Comparison of biological activities of human antithrombins with high-mannose or complex-type nonfucosylated N-linked oligosaccharides
Source: Glycobiology. 2016 Jan 7;26(5):482–92. doi: 10.1093/glycob/cww001 (PMC4813732; doi:10.1093/glycob/cww001)
Supplement: Supplementary Data [file supp_26_5_482__index.html]

Comparison of biological activities of human antithrombins with high-mannose or complex- type nonfucosylated N-linked oligosaccharides — Comparison of biological activities of human antithrombins with high-mannose or complex-type nonfucosylated N-linked oligosaccharides — Comparison of biological activities of human antithrombins with high-mannose or complex-type nonfucosylated N-linked oligosaccharides — Supplementary Data 

# Comparison of biological activities of human antithrombins with high-mannose or complex-type nonfucosylated N-linked oligosaccharides

## Supplementary Data

Supplementary Data

- Supplementary Data - Doc file
