# Supplementary material for: Comparison of biological activities of human antithrombins with high-mannose or complex-type nonfucosylated N-linked oligosaccharides
Source: Glycobiology. 2016 Jan 7;26(5):482–92. doi: 10.1093/glycob/cww001 (PMC4813732; doi:10.1093/glycob/cww001)
Supplement: Supplementary Data [file supp_cww001_cww001supp.doc]

**Supplementary materials**

**Table S1**

The content of the aggregates and latent form in the purified rhATs

Samples ^a^Aggregates (%) ^b^Latent form (%)

phAT 0.4 10.2

rhAT-Manα n.d. 1.8

rhAT-Manβ 0.4 0.5

rhAT-Comα 0.7 0.8

rhAT-Comβ n.d. n.d.

^a^The aggregates were detected by size-exclusion chromatography.

^b^The latent form was detected by hydrophobic interaction chromatography.

n.d., not detected.

**Table S2**

The content of the α-form in the purified rhATs

Samples ^a^α-form (%)

phAT 97

rhAT-Manα 100

rhAT-Manβ n.d.

rhAT-Comα 100

rhAT-Comβ n.d.

^a^The α-form was detected by hydroxyapatite chromatography.

n.d., not detected.
